# Long-term humoral and cellular immunity after primary SARS-CoV-2 infection: a 20-month longitudinal study

**DOI:** 10.1186/s12865-023-00583-y

**Published:** 2023-11-16

**Authors:** Astrid Korning Hvidt, Huaijian Guo, Rebecca Andersen, Stine Sofie Frank Lende, Line Khalidan Vibholm, Ole Schmeltz Søgaard, Marianne Hoegsbjerg Schleimann, Victoria Russell, Angela Man-Wei Cheung, Eustache Paramithiotis, Rikke Olesen, Martin Tolstrup

**Affiliations:** 1 Department of Infectious Diseases, Aarhus University Hospital, Aarhus, Denmark; 2 Department of Clinical Medicine, Aarhus University, Aarhus, Denmark; 3 CellCarta, Montreal, QC Canada; 4 Toronto General Hospital Research Institute, University Health Network, Toronto, ON Canada; 5 Department of Medicine, University Health Network, Toronto, ON Canada; 6 Department of Medicine, University of Toronto, Toronto, ON Canada; 7 Institute of Health Policy, Management and Evaluation, University of Toronto, Toronto, ON Canada

**Keywords:** SARS-CoV-2, Infection, Antigen-specific T cells, Antibodies, Vaccine, Immune durability

## Abstract

**Background:**

SARS-CoV-2 remains a world-wide health issue. SARS-CoV-2-specific immunity is induced upon both infection and vaccination. However, defining the long-term immune trajectory, especially after infection, is limited. In this study, we aimed to further the understanding of long-term SARS-CoV-2-specific immune response after infection.

**Results:**

We conducted a longitudinal cohort study among 93 SARS-CoV-2 recovered individuals. Immune responses were continuously monitored for up to 20 months after infection. The humoral responses were quantified by Spike- and Nucleocapsid-specific IgG levels. T cell responses to Spike- and non-Spike epitopes were examined using both intercellular cytokine staining (ICS) assay and Activation-Induced marker (AIM) assay with quantification of antigen-specific IFNγ production. During the 20 months follow-up period, Nucleocapsid-specific antibody levels and non-Spike-specific CD4 + and CD8 + T cell frequencies decreased in the blood. However, a majority of participants maintained a durable immune responses 20 months after infection: 59% of the participants were seropositive for Nucleocapsid-specific IgG, and more than 70% had persisting non-Spike-specific T cells. The Spike-specific response initially decreased but as participants were vaccinated against COVID-19, Spike-specific IgG levels and T cell frequencies were boosted reaching similar or higher levels compared to 1 month post-infection. The trajectory of infection-induced SARS-CoV-2-specific immunity decreases, but for the majority of participants it persists beyond 20 months. The T cell response displays a greater durability. Vaccination boosts Spike-specific immune responses to similar or higher levels as seen after primary infection.

**Conclusions:**

For most participants, the response persists 20 months after infection, and the cellular response appears to be more long-lived compared to the circulating antibody levels. Vaccination boosts the S-specific response but does not affect the non-S-specific response. Together, these findings support the understanding of immune contraction, and with studies showing the immune levels required for protection, adds to the knowledge of durability of protection against future SARS-CoV-2.

**Supplementary Information:**

The online version contains supplementary material available at 10.1186/s12865-023-00583-y.

## Background

In late 2019 Severe Acute Respiratory Syndrome Coronavirus 2 (SARS-CoV-2) emerged, causing the Coronavirus Disease 2019 (COVID-19) pandemic. Now, three years later, the World Health Organisation (WHO) has declared that SARS-CoV-2 no longer constitutes a public health emergency of international concern, but infection remains an ongoing health issue and SARS-CoV-2 variant waves still have a great impact on public health [1]. The humoral and cellular immune responses elicited by SARS-CoV-2 infection can lead to viral clearance, protect against severe disease, and can generally limit viral spread [2–4]. High antibody titers have been correlated with protection against different variants of concern but cannot provide definitive immunity [5–7]. The T cell response is also essential for both achieving viral clearance and limiting disease severity, but the role of the T cell responses is less understood [3, 8].

Early in the pandemic, and following the approval of Spike-based COVID-19 vaccines, great efforts have been put into investigating immunodominant peptide domains within SARS-CoV-2, and a great portion has been identified in the Spike (S) protein [9–12]. Yet, other epitopes outside the S-protein have also showed great potential [9, 10, 12–14], but as vaccines were rolled out the focus on non-S-specific immune characterization declined. The non-S-specific humoral and cellular responses grant an understanding of the adaptive immune contraction over time regardless of vaccination, while the S-specific response enables tracking of the temporal booster effect of vaccination. A decrease in Nucleocapsid (N)-specific antibody levels over time has previously been shown independent of vaccination [10, 15]. The levels of S-specific T cells are also well described, with an upregulation upon infection and vaccination [16, 17]. Some studies have also characterized non-S-specific T cells following infection [15, 17], but there is a lack of longitudinal studies investigating the long-term dynamics of the natural immune response after primary infection, which will help us understand the expected longevity of the T cell response, and thus the necessity of a continued vaccine effort.

Therefore, we selected 93 SARS-CoV-2 recovered participants based on having completed all longitudinal visits, from the previously described CoroNAT cohort [18, 19]. We characterized their adaptive immune responses 1 month after initial SARS-CoV-2 infection and at 3 consecutive time points during a 20-month follow-up. We performed in-depth analyses of the adaptive immune responses involving longitudinal T cell responses towards both S and non-S peptides as well as S-specific and N-specific antibody levels.

## Results

**Fig. 1:**
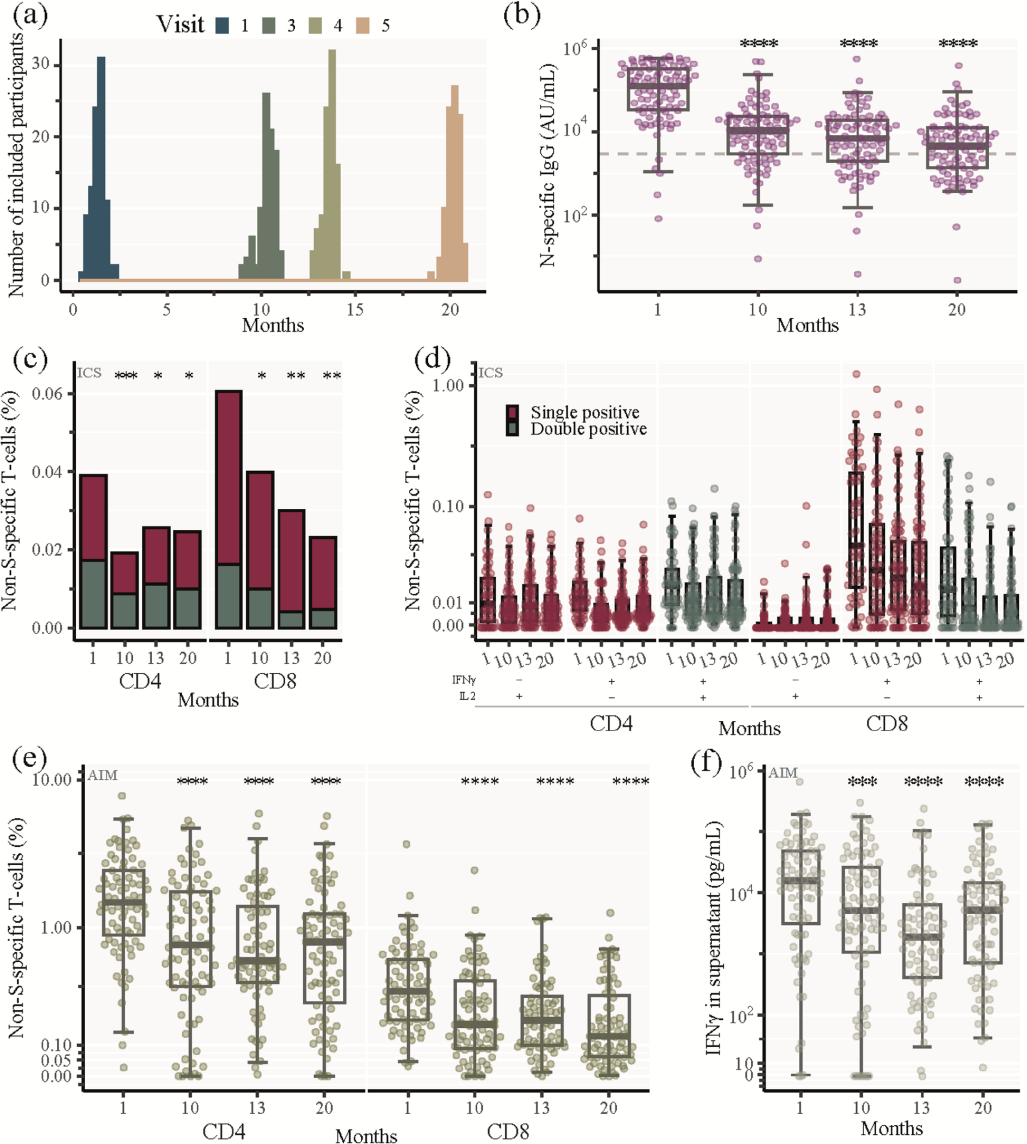
SARS-CoV-2 non-Spike-specific longitudinal immune response. **a** Sampling time of the 93 participants for each visit. Zero months represents the time of infection defined as positive PCR test. **b** SARS-CoV-2 nucleocapsid (N)-specific IgG analysed by Mesoscale. Being seropositive was defined as IgG levels above 3000 AU/mL (dashed line). **c**, **d** Percentage of SARS-CoV-2 non-spike (S)-specific CD4 + and CD8 + memory T cells analysed by ICS. **c** Total SARS-CoV-2 non-S-specific CD4 + and CD8 + memory T cells at indicated time points. IFNγ only and IL2 only producing cells: red bar, IFNγ and IL2 double producing cells: green bar. Stacked bars represent median values. **d** Percentage of SARS-CoV-2 non-S-specific CD4 + and CD8 + memory T cells producing either IL2 only (red, left panel), IFNγ only (red, middle panel), or co-producing IFNγ and IL2 (green, right panel) at each visit. **e**, **f** Percentage of SARS-CoV-2 non-S-specific CD4 + and CD8 + T cells analysed by AIM. **e** Total SARS-CoV-2 non-S-specific CD4 + and CD8 + T cells at indicated time points, i.e., cells expressing 2 or 3 activation induced markers (CD69, OX40 or 41BB). **f** IFNγ production by SARS-CoV-2 non-S-specific cells measured in the supernatant harvested from the cell stimulations in the AIM assay (Analysed by mesoscale). **b**, **d**, **e**, **f** Box and whisker plots show median values ± IQR and error bars indicate 95% CI. Statistical comparisons were performed using Friedman test and Wilcoxon unpaired signed-ranks test adjusted using Bonferroni with visit 1 as a reference. *P ≤ 0.05, **P < 0.01, ***P < 0.001, ****P < 0.0001, no asterisk indicates non-significance

The 93 participants from the CoroNAT cohort had completed 4 study visits at approximately 1, 10, 13 and 20 months post primary SARS-CoV-2 infection, respectively (Fig. 1A). The demographics of the participants are shown in Table 1. Briefly, 54% of the cohort were males, and the median age was 48 years (range 20–68 years). The majority of participants (84%) experienced mild SARS-CoV-2 infection (disease severity group 1 + 2), while 16% were hospitalized (disease severity group 3 + 4). COVID-19 vaccines became available during the follow-up period, and the participants received vaccinations according to national guidelines. The vaccine status of the participants can be seen in Supp. Table 1. At 10 months post-infection, 9.7% had received their first vaccine dose, at month 13, 33% had received 1 or 2 vaccine doses and at month 20, 99% had received one, two or three vaccine doses [18, 19].

**Table 1:** Demographics at time of inclusion

	*N* = 93
**Sex, n (%)**	
Female	43 (46%)
Male	50 (54%)
**Age (years), Median [IQR]**	48 [42, 55]
**Comorbidities (No. of comorbidities), n (%)**	
> 1 Comorbidities	40 (43%)
No Comorbidities	53 (57%)
**BMI index, n (%)**	
Normal	45 (48%)
Overweight	28 (30%)
Obese	20 (22%)
**Disease severity group, n (%)**	
1	9 (9.7%)
2	69 (74%)
3 + 4	15 (16%)

Table showing demographics at time of inclusion. Disease severity group is divided as follows: 1) Home/outpatient not experiencing any limitations in daily life; 2) Home/outpatient, certain limitations in daily activity level (e.g., fever, bedridden during illness); 3+4) All hospitalized patients regardless of need for supplemental oxygen treatment, and/or ICU admission

### SARS-CoV-2 non-spike-specific longitudinal immune response

To investigate the non-vaccine related immune response towards primary SARS-CoV-2 infection we measured SARS-CoV-2 nucleocapsid (N)-specific IgG as well as the frequency of antigen-specific T cells targeting nucleocapsid and other non-S elements. At 1 month post-infection, the median level of N-specific IgG was 127,556 AU/mL which then declined gradually over the course of the study to 4,542 AU/mL after 20 months (Fig. 1B).

We characterized the non-vaccine related cellular response using two separate polychromatic flow cytometry assays, ICS and AIM, after ex vivo stimulation of PBMCs with a non-S peptide pool (34 immunodominant peptides outside the S protein). We first characterized CD4 + and CD8 + memory differentiation in naïve, central memory, effector memory and terminal differentiated subsets, using the ICS assay, and found only minor changes over the 4 visits (Supp. Figure 1). We then measured IFN-γ and IL2 expression in unfractionated memory CD4 + and CD8 + T cells, i.e., central memory, effector memory and terminally differentiated subsets combined (Supp. Figure 2). At 1 month post-infection there were high levels of both CD4 + and CD8 + non-S-specific memory T cells (Median: 0.039% and 0.061%, respectively) (Fig. 1C). After 10, 13 and 20 months post-infection the frequency of non-S-specific cells significantly decreased to 0.019% (*p* = 0.0007); 0.026% (*p* = 0.02) and 0.025% (*p* = 0.01), respectively for CD4 + memory T cells, and 0.040% (*p* = 0.03); 0.030% (*p* = 0.004) and 0.023% (*p* = 0.001), respectively, for CD8 + memory T cells (Fig. 1C).

The cytokine profile of the memory CD4 + T cells consisted of IL2 single (IL2 + /IFNγ-), IFNγ single (IL2-/IFNγ +) and IL2/IFNγ double (IL2 + /IFNγ +) producing cells. The highest frequency of each population was observed at 1 month post-infection, (Median: 0.0093%; 0.012%; and 0.018% for IL2 + / IFNγ-; IFNγ + /IL2-; and IL2 + /IFNγ + CD4 + T cells, respectively) (Fig. 1D). In contrast, the SARS-CoV-2 memory CD8 + T cells consisted primarily of IFNγ single producing cells and to a lesser degree IL2/IFNγ double producing cells. The highest frequency of each population was observed at visit 1 (Median: 0.045% and 0.016% for IFNγ + /IL2- and IL2 + /IFNγ + CD8 + T cells, respectively) (Fig. 1D).

Evaluation of non-S-specific CD4 + and CD8 + T cells using AIM revealed higher levels of non-S-specific T cells than measured by ICS. One month after infection the median frequencies of non-S-specific T cells using AIM were 1.48% for CD4 + T cells and 0.35% for CD8 + T cells. We observed a contraction at 10 months post-infection (median: 0.76% (*p* < 0.0001) for CD4 + T cells and 0.18% (*p* < 0.0001) and CD8 + T cells) after which the response remained stable throughout the 20-month follow-up period (Fig. 1E). From the PBMCs stimulated with the non-S peptide pool for the AIM assay, we collected the supernatant and measured the IFNγ-secretion. We found the highest median IFNγ-production 1 month after infection (15,806 pg/mL), which significantly decreased at the subsequent study visits (5,144 pg/mL (*p* = 0.0004); 1,885 pg/mL (*p* < 0.0001) and 5,214 pg/mL (*p* < 0.0001) at month 10, 13 and 20, respectively).

Collectively, we found high levels of both N-specific antibodies and non-S-specific CD4 + and CD8 + T cells in response to primary SARS-CoV-2 infection. Ten months after infection, both the circulating antibody levels and the T cell responses had contracted significantly. However, in the following period up to 20 months, both the humoral and cellular immune responses decreased at a slower rate.

### Durability of SARS CoV-2 infection-induced immunity

**Fig. 2:**
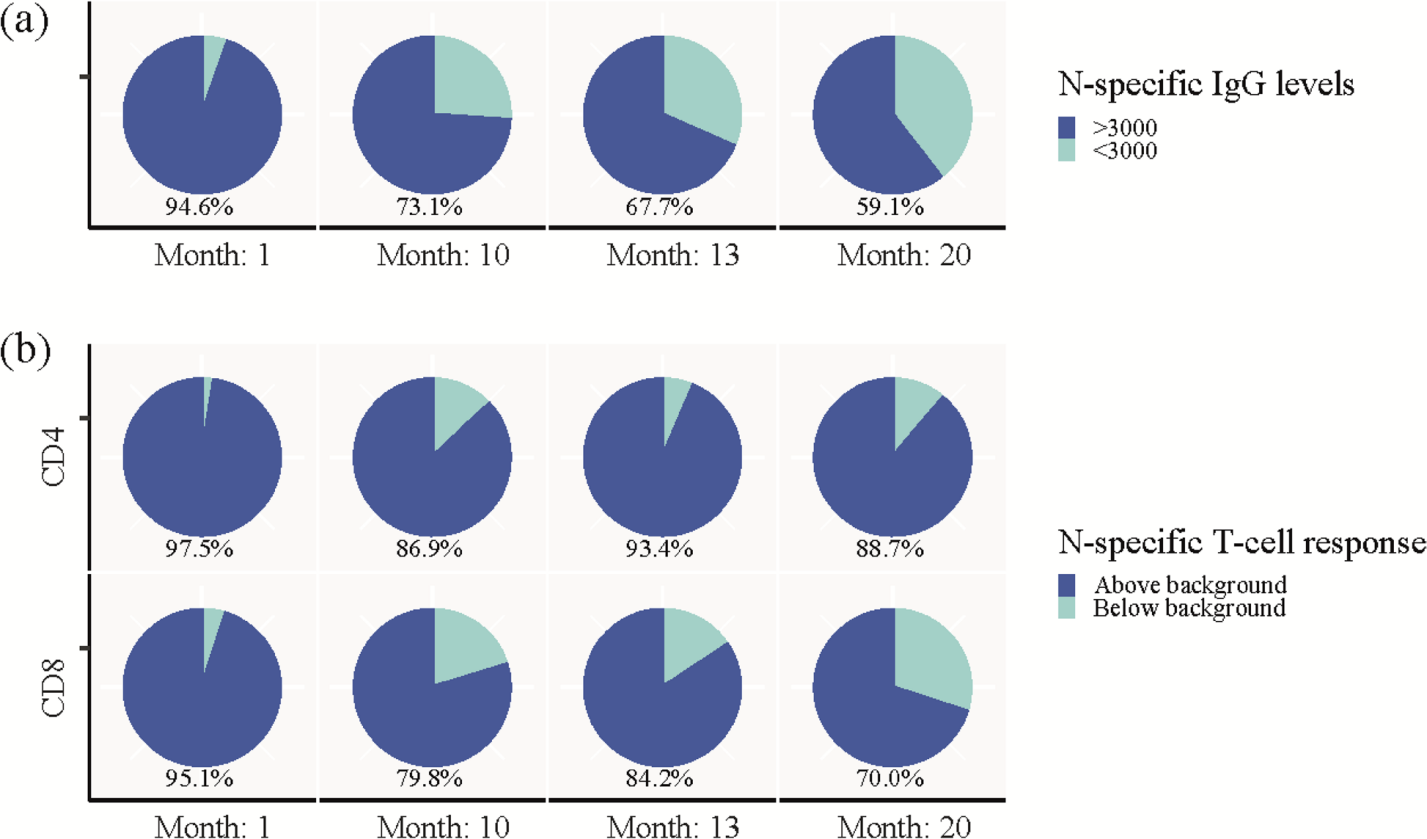
Durability of SARS-CoV-2 infection-induced immunity. **a** Pie charts showing percentage of participants with SARS-CoV-2 nucleocapsid-specific IgG above (dark blue) and below (light blue) 3000 AU/mL at each visit. **b** Pie charts showing percentage of participants with SARS-CoV-2 non-spike-specific CD4 + (top panel) and CD8 + (lower panel) T cells above (dark blue) or below (light blue) background response analysed by AIM

To evaluate the durability of the SARS-CoV-2 immune response, we defined seropositivity as N-specific IgG levels above 3000 AU/mL. We defined a positive AIM response as non-S-specific T cells as levels above 0.107% for CD4 + T cells and 0.078% for CD8 + T cells, in accordance with Dietz el al. [16]. One month post-infection, 94.6% of the participants were seropositive for N-specific IgG. During the study, the number of N-specific seropositive participants decreased to 73.1%, 67.7% and 59.1% at the 10-, 13-, and 20-month study visits, respectively (Fig. 2A). At 1 month post-infection, 97.5% of participants had a positive response for non-S-specific CD4 + T cells, and 88.7% of participants maintained a positive response after 20 months. For non-S-specific CD8 + T cells, 95% of participants had a positive response at the 1-month visit and 70% of participants sustained a positive response at 20 months post-infection (Fig. 2B).

### SARS-CoV-2 S-specific longitudinal immune response

**Fig. 3:**
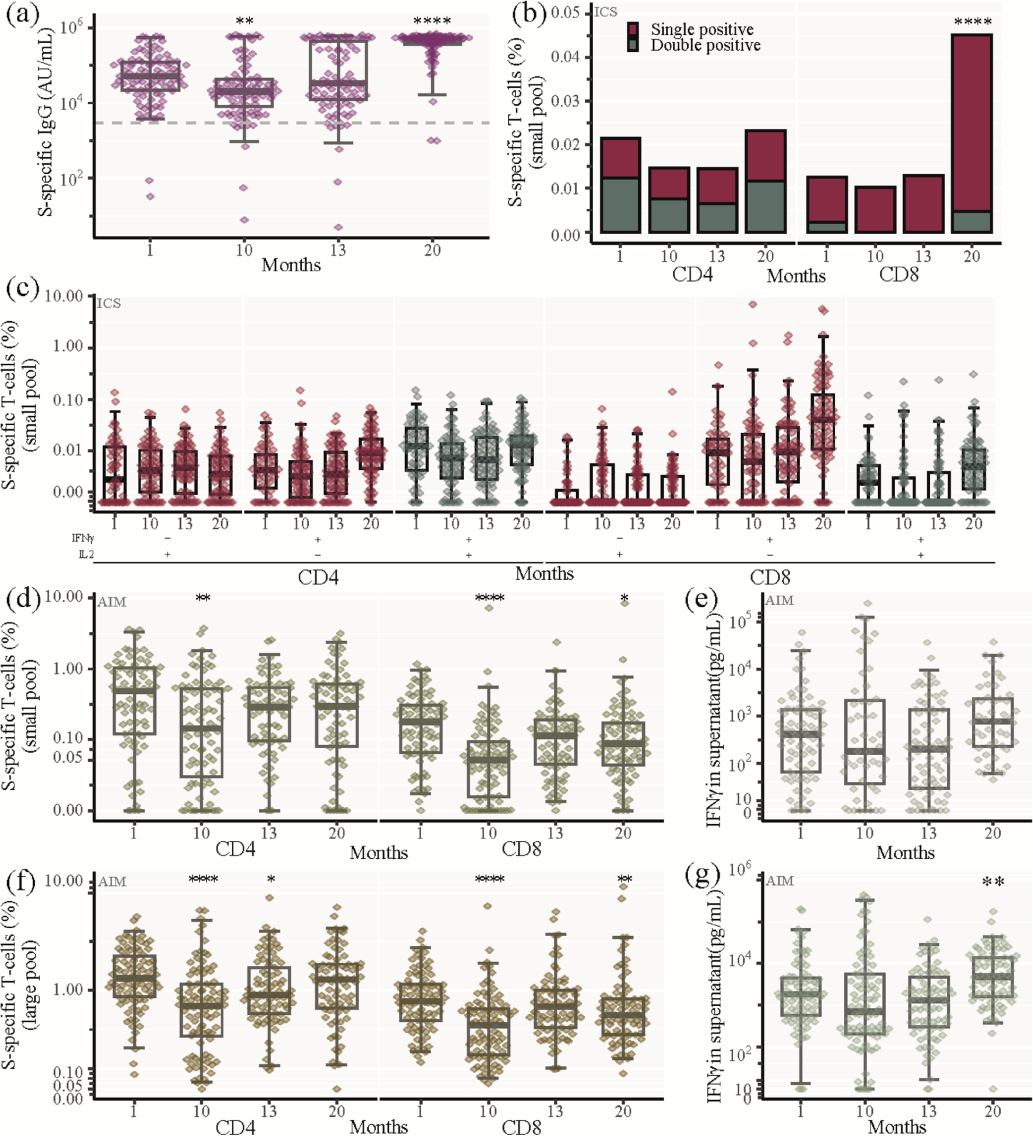
SARS-CoV-2 Spike-specific longitudinal immune response. **a** SARS-CoV-2 spike (S)-specific IgG analysed by Mesoscale. Being seropositive was defined as IgG above 3000 AU/mL (dashed line). **b**, **c** Percentage of SARS-CoV-2 S-specific CD4 + and CD8 + memory T cells analysed by ICS after stimulation with the S-small peptide pool. **b** Total SARS-CoV-2 S-specific CD4 + and CD8 + T memory cells at indicated time points. IFNγ only and IL2 only producing cells: red bar, IFNγ and IL2 double producing cells: green bar. Stacked bars represent median values. **c** Percentage of SARS-CoV-2 S-specific CD4 + and CD8 + memory T cells producing either IL2 only (red, left panel), IFNγ only (red, middle panel), or co-producing IFNγ and IL2 (green, right panel) at each visit. **d-g** Percentage of SARS-CoV-2 S-specific CD4 + and CD8 + T cells analysed by AIM after stimulation with the S-small (**d**, **e**) or the large (**f**, **g**) peptide pool, i.e., cells expressing 2 or 3 activation induced markers (CD69, OX40 or 41BB). **d** SARS-CoV-2 S-specific CD4 + and CD8 + T cells at indicated time points (S-small pool). **e** IFNγ production by SARS-CoV-2 S-specific cells (S-small pool) (Analysed by mesoscale). **f** SARS-CoV-2 S-specific CD4 + and CD8 + T cells at indicated time points (S-large pool). **g** IFNγ production by SARS-CoV-2 S-specific cells (S-large pool) (Analysed by mesoscale). **a**, **c**, **d-g** Box and whisker plots show median values ± IQR and error bars indicate 95% CI. Statistical comparisons were performed using Friedman test and Wilcoxon unpaired signed-ranks test adjusted using Bonferroni with visit 1 as a reference. *P ≤ 0.05, **P < 0.01, ***P < 0.001, ****P < 0.0001, no asterisk indicates non-significance

Next, we assessed the strength of the humoral immunological memory response elicited by vaccination. At 1 month post-infection, the median level of S-specific IgG was 51,016 AU/mL and had declined to 20,551 AU/mL at 10 months post-infection **(**Fig. 3A). As the majority of participants received COVID-19 vaccination median S-specific IgG levels increased to 493,232 AU/mL at month 20 post-infection demonstrating a strong memory response to vaccination with a tenfold increase in IgG levels compared to the levels immediately after infection (Fig. 3A).

To characterize the cellular immunological memory response to vaccination, we first used ICS with ex vivo stimulation of PBMCs with a S-small peptide pool (11 immunodominant S peptides). One month post-infection, both CD4 + and CD8 + S-specific memory T cells (Median: 0.021% and 0.012%, respectively) were detected. These levels declined slightly by 10 and 13 months post-infection (Fig. 3B). At 20 months post-infection, where 99% of the participants had received at least one vaccine dose, the percentage of CD4 + S-specific memory T cells had only increased slightly compared to 1 month post-infection. On the other hand, there was a fourfold increase of CD8 + S-specific memory T cells (Median: 0.045%, *p* < 0.0001) at the 20-month visit (Fig. 3B). The cytokine profile of the S-specific memory T cells consisted, for CD4 + memory T cells of IL2 + /IFNγ-, IL2-/IFNγ + and IL2 + /IFNγ + producing cells, while the CD8 + memory response consisted primarily of IL2-/IFNγ + producing cells (Fig. 3C).

Additionally, we evaluated the S-specific T cell immunological memory response using the AIM assay after stimulation with either the S-small peptide pool (11 selected peptides) (Fig. 3D, E) or an S-large peptide pool (315 overlapping peptides spanning the entire S protein (Fig. 3F, G). At 1-month post-infection, we observed a median of 0.49% and 0.18% for CD4 + and CD8 + T cells, respectively, using the S-small pool. Using the S-large pool the levels were greater with a median of 1.34% and 0.77% for CD4 + and CD8 + T cells, respectively (Fig. 3D + F). At 10 months post-infection, there was a significant decrease of both S-specific CD4 + and CD8 + T cells (Median: S-small pool: 0.11% and 0.05% for CD4 + and CD8 + T cells, respectively; S-large pool: 0.69% and 0.42% for CD4 + and CD8 + T cells, respectively). At the 20-month follow-up, most participants (92 of 93) had received COVID-19 vaccinations and the frequency of S-specific CD4 + T cells had increased to levels comparable to 1 month after infection (Fig. 3D + F). The frequency of S-specific CD8 + T cells had also increased at the 20-month follow-up relative to the month 10 time-point, but the response did not reach the same level as 1 month post-infection (Fig. 3D + F). We observed a 2.5-fold increase in the S-specific IFNγ-production at 20 months post-infection relative to 1 month post-infection (Fig. 2G), consistent with the increase of IFN producing CD8 + T cells measured by ICS. Lastly, at 10 months post-infection, there was a significant decrease of S-specific IL4 production, which was followed by an increase at 20 months post-infection, to levels comparable to 1 month after infection (Supp. Figure 3).

**Fig. 4:**
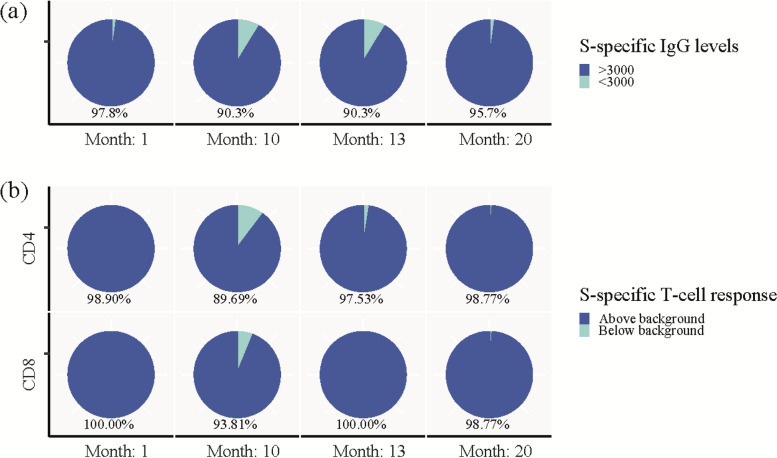
Population frequency of Spike-specific immune responses. **a** Pie charts showing percentage of participants with SARS-CoV-2 spike (S)-specific IgG above (dark blue) and below (light blue) 3000 AU/mL at each visit. **b** Pie charts showing percentage of participants with SARS-CoV-2 S-specific CD4 + (top panel) and CD8 + (lower panel) T cells above (dark blue) or below (light blue) background response analysed by AIM after stimulation with the S-large pool

As previously described, we defined being seropositive as S-specific IgG levels above 3000 AU/mL [20, 21] and a positive AIM response as S-specific CD4 + and CD8 + T cells as levels above 0.107% and 0.078%, respectively [16]. We used this to further characterise the impact of vaccination on the S-specific immune response. One month after infection 97.8% were seropositive for S-specific IgG, which decreased to 90.3% at 10 months. Twenty months post-infection, 99% of participants had received vaccination and 95.7% were seropositive (Fig. 4A). With regards to T cell responses, we observed that at 1 month post-infection 98.9% and 100.0% had a S-specific CD4 + and CD8 + T cell response, respectively, while at ten months post-infection, 89.7% and 93.8% had maintained a S-specific CD4 + and CD8 + T cell response, respectively. At 20 months post-infection the frequency of participants with a S-specific T cell response had increased to 98.8% for both T cell subsets (Fig. 4B).

### Evaluation of SARS-CoV-2 vaccination on humoral and cellular immunity

To perform a direct evaluation of the immune-boosting effect of 2 vaccinations after primary infection, we selected a subset of patients, who had received their first 2 vaccinations between two subsequent visits (*n* = 65, Supp. Table 2), and compared the humoral and cellular immunological memory responses.

**Fig. 5:**
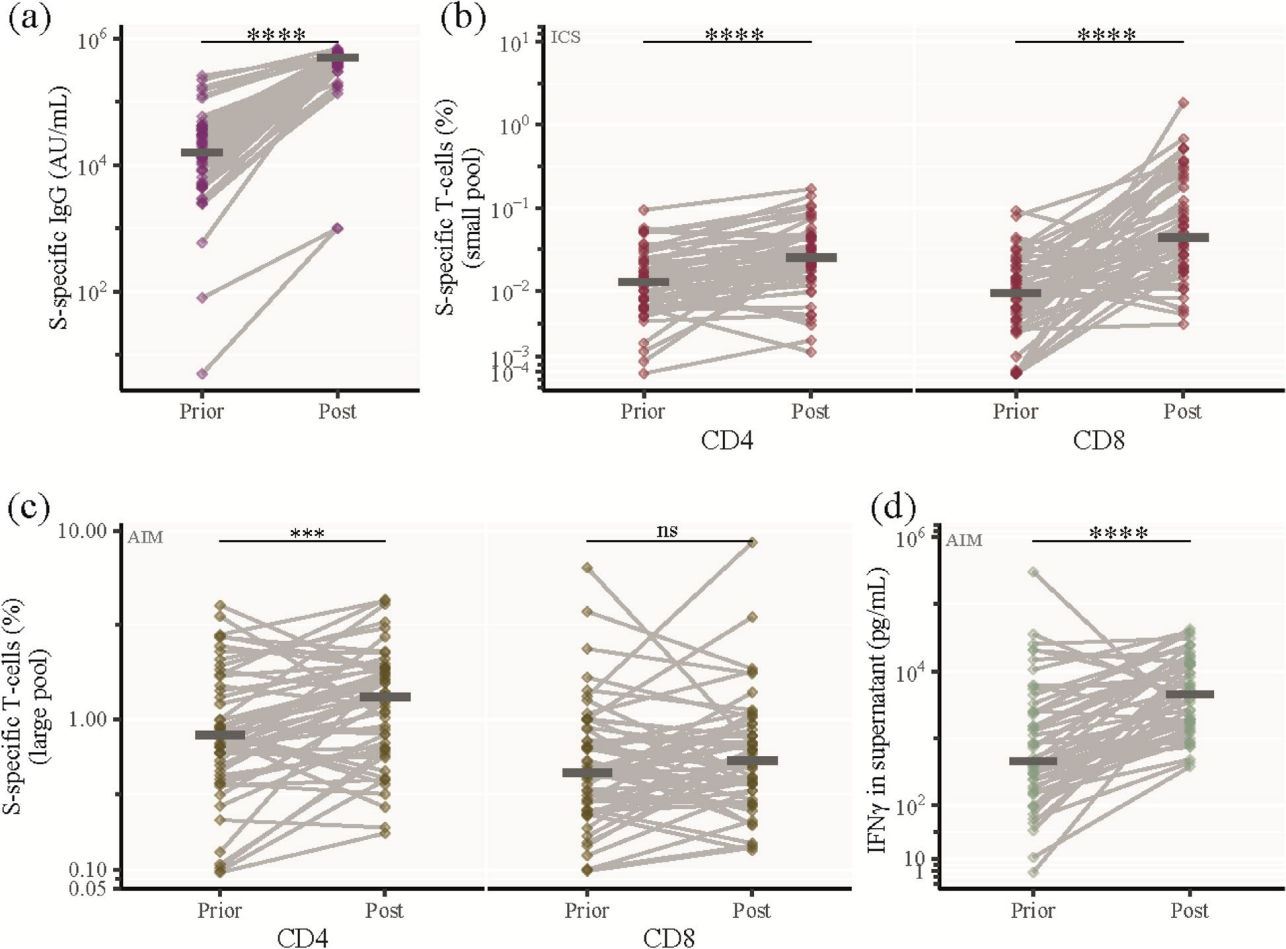
Evaluation of SARS-CoV-2 vaccination on humoral and cellular immunity. Analysis of a subset of 65 patients who received their first 2 vaccinations between two subsequent visits (Prior: A visit where the participant had not been vaccinated, Post: The subsequent visit, where the participant had received 2 vaccinations). **a** SARS-CoV-2 S-specific IgG levels (**b**) Percentage of SARS-CoV-2 S-specific CD4 + and CD8 + memory T cells analysed by ICS after stimulation with the S-small peptide pool. **c** Percentage of SARS-CoV-2 S-specific CD4 + and CD8 + T cells analysed by AIM after stimulation with the S-large pool. **d** IFNγ production by SARS-CoV-2 S-specific cells (S-large pool). Horizontal line shows median. Statistical comparisons were performed using Wilcoxon unpaired signed-ranks test adjusted using Bonferroni. *P ≤ 0.05, **P < 0.01, ***P < 0.001, ****P < 0.0001

Prior to vaccination, the median level of S-specific IgG was 16,003 AU/mL which increased to 501,794 AU/mL after two vaccinations (*p* < 0.0001) (Fig. 5A). Using the ICS assay, we also detected a significant increase in both S-specific CD4 + and CD8 + memory T cells from 0.013% to 0.025% (*p* < 0.0001) and 0.009% to 0.044% (*p* < 0.0001), respectively (Fig. 5B). When measuring S-specific T cells using the AIM assay, there was no increase in CD8 + T cells, but we found a significant increase in both CD4 + T cells (0.82% to 1.32%, *p* = 0.0007) and IFNγ production (454 pg/mL to 4,558 pg/mL, *p* < 0.0001) (Fig. 5C, D, Supp. Figure 4A, B). Additionally, we investigated the non-S-specific response before and after vaccination, but we did not identify any significant increases in N-specific IgG levels or non-S-specific T cells (Supp. Figure 4B-F).

## Discussion

In this study, we investigated the longitudinal humoral and cellular response trajectories following primary SARS-CoV-2 infection up to 20 months post-infection. One month after primary infection, more than 94% of all participants seroconverted for both N- and S-specific IgG. Both non-S- and S-specific T cells were also detected in more than 95% of participants, which demonstrated the adaptive immune response to infection. Over the following 20 months after infection, both the levels of non-S-specific humoral and cellular immunity decreased but remained detectable in the majority of participants implying efficient long-term immune memory formation. Greater proportions of participants had a quantifiable persisting T cell response compared to persisting circulating antibody levels when evaluating the responses to non-S antigens. This suggests very long-lasting cellular immune memory similar to what has been observed for the first SARS-CoV-1 outbreak, where antigen specific T cells could be detected in infected individuals for more than a decade post-infection[22, 23]. The S-specific humoral and cellular response also decreased in the first 10 months ensuing the initial infection, but a robust S-specific memory response was then elicited upon vaccination.

These findings confirm results from multiple other studies investigating the longitudinal SARS-CoV-2 immune response, showing a general decline of both the humoral and cellular SARS-CoV-2-specific immune response after 12 months [10, 17, 24] and a strong vaccine-induced S-specific memory response [25]. However, this study includes data on both humoral and cellular immunity 20 months after primary SARS-CoV-2 infection, thus providing new key insights into the longevity of the adaptive immune memory. Additionally, we have investigated both S- and non-S-specific T cells, allowing us to differentiate between the naturally contracting immunity after primary infection and the following vaccine-induced memory response. Gittelman et al. has used T cell receptor (TCR) sequencing to evaluate the breadth and depth of SARS-CoV-2-specific TCR towards both S and other viral proteins. They found a high frequency of SARS-CoV-2 associated TCRs 15 months after infection with increased clonal breadth and depth for the S protein in participants who received vaccination, while they did not see such an increase for the non-S proteins [26]. Our data substantiate the findings by Gittelman et al. both in terms of detecting long-term cellular responses as well as clearly being able to distinguish between the course of immunity induced by primary infection (non-S immunity) versus S-specific immunity boosted by vaccination. Further, we complement this data by providing a more detailed description of the functionality of the T cell response, by measuring the frequency of SARS-CoV-2-specific T cells using two different but complementary flow cytometric T cell assays (ICS and AIM), which includes the antigen-specific production of IFNγ and IL2 in response to SARS-CoV-2 peptide stimulation. These findings are in alignment, showing a natural durability of a non-S-specific response even after 15 and 20 months in the majority of participants.

Even though a reduction of the SARS-CoV-2-specific immune response was observed, the non-S-specific response persisted even after 20 months. During this follow-up period (April 2020 – November 2021) the level of community acquired infections remained relatively low in Denmark with population cumulative infection estimates at 5% [5, 27]. None of the participants reported re-infection of SARS-CoV-2 during the follow-up period, but undiagnosed asymptomatic infections cannot be ruled out. However, given the low level of population infections, the relatively few undiagnosed cases would not interfere with our conclusions. Other studies have shown an increase of antigen-specific T cells without known infection or seroconversion [17, 28–32]. Cross-reactivity to other human coronaviruses does not seem prominent, but some specificity may be preserved in epitopes in the Nucleocapsid region [13, 33]. Further investigations regarding non-S T cell responses may prove helpful in terms of expanding the protection against future SARS-CoV-2 variant waves.

We performed two flow cytometry assays, ICS and AIM, to characterize SARS-CoV-2-specific T cells induced by infection. Due to the nature of the different primary analytes of two assays, the ICS provides an approximate equal detection of the antigen-specific CD4 + and CD8 + T cell response because of the easily detectable intracellular IFNγ and IL2 in each subset, while some of the activation-induced markers used in the AIM assay are more prevalent on CD4 + T cells favouring detection of antigen-specific CD4 + T cells.

This phenomenon explains, why we detected higher levels of CD8 + versus CD4 + non-S-specific T cells at the 1-month time-point using ICS, while detecting higher levels of CD4 + versus CD8 + non-S-specific T cells using AIM. However, since the AIM assay is relying on more broadly expressed surface markers and is not limited to a few selected cytokines, we were still able to detect significantly higher levels of both antigen-specific CD4 + and CD8 + T cells using the AIM assay versus, verifying that AIM is a very useful addition to ICS when estimating the total levels of antigen-specific T cells. Further, when measuring IFNγ from the AIM supernatants, we observed a response pattern similar to that of ICS IFNγ expressing CD8 + T cells, demonstrating the response of antigen-specific CD8 + T cells. After vaccination we observed an induction of S-specific T cells to similar or higher levels as compared to 1 month after infection. Previously, Grifoni et al. compiled various epitope data and showed that CD8 + respond to a high number of immunogenic epitopes spanning the total S protein [34]. Looking at the ICS, the vaccine induced response is especially prominent for CD8 + T cells as well as for the IFNγ production from the AIM supernatants, where levels were increased more than twofold compared to 1 month post-infection, supporting a great vaccine-induced S-specific CD8 + T cell response.

## Conclusions

This study shows long-term trajectories of both humoral and cellular immunity after SARS-CoV-2 infection. For most participants, the response persists 20 months after infection, and the cellular response appears to be more long-lived compared to the circulating antibody levels. Vaccination clearly boosts the S-specific response but does not affect the non-S-specific response. Together, this supports the understanding of immune contraction, and with studies showing the immune levels required for protection, adds to the knowledge of durability of protection against future SARS-CoV-2.

## Methods

### Study design and sample collection

Participants were enrolled at the Department of Infectious Diseases at Aarhus University Hospital, Denmark from April 3rd to July 9th 2020. Participants who had completed a study visit at 1, 10, 13, and 20 months after infection were included (*n* = 93). All participants had recovered from a PCR-verified SARS-CoV-2 infection. The study was approved by The National Health Ethics Committee (#1–10-72–76-20) and the Danish Data Protection Agency (case number not applicable). All participants provided informed written consent prior to any study activities. A more detailed description of the cohort has been performed by Nielsen et al. and Vibholm et al. [18, 19].

### Quantification of SARS-CoV-2 IgG

Serum levels of SARS-CoV-2 Spike- and Nucleocapsid-specific IgG levels were measured using Meso Scale Discovery (MSD) platform (Meso Scale Diagnostics LLC, Maryland, USA) panel 2 IgG kit (K15383U-2). The assays were performed according to the manufacturer’s protocol with a serum dilution of 1:5,000 for IgG measurements. Plates were read on a MESO QuickPlex SQ 120 reader. Raw data were processed by Discovery Workbench 4.0 Software. Quantifications were reported in arbitrary units per mL (AU/mL).

We have previously utilized this analysis to evaluate the variation of Nucleocapsid antibody levels in a large Danish nation-wide prospective vaccination cohort and defined a cut-off for seroconversion to be a value above 3000 AND a > twofold increase above baseline [20, 21]. Using these parameters, we observed positive seroconversion in 96% of participants with a verified PCR-diagnosis [35]. In the present evaluation all individuals had been infected and therefore, seroconversion could not be defined as a twofold increase like in our previous publication. Instead, we have used only the 3000 AU/mL cut-off for the definition of seroconversion to evaluate the level of persistence of nucleocapsid antibodies.

### Evaluation of antigen-specific T cells using flow cytometry

Antigen-specific T cells were measured by the two methods i.e., Activation Induced Marker assay (AIM) and Intracellular Cytokine Stain assay (ICS). For both AIM and ICS cryopreserved PBMCs were thawed, washed, and rested at 37˚C/5% CO2 for 3–4 h. Cells were then plated into wells of a 96-well plate, at a total of 1 × 106 cells per well and stimulated with SARS-CoV-2 peptide pools, containing a mix of peptides specific for both CD4 + and CD8 T + cells. The following stimulations were used for the AIM/ICS assays: No exogenous stimulation with Dimethyl sulfoxide (DMSO) as negative control (AIM/ICS), a S peptide large pool (S-large) consisting of 315 overlapping peptides (JPT peptides, #PM-WCPV-S-2, Swiss-Prot ID: P0DTC2, REF PMID: 32,015,508)(AIM); an S peptide small pool (S-small) consisting of 11 immunodominant S peptides [8, 9, 12, 13, 36, 37] (Supp. Table 3) (JPT peptides, custom order)(AIM/ICS); and a non-S peptide pool (non-S) consisting of 34 immunodominant peptides outside the S protein [8, 9, 12, 13, 36, 37] (Supp. Table 3) (JPT peptides, custom order) (AIM/ICS) used at a final concentration of 2 μg/mL of each peptide. Upon antigen stimulation, antigen-specific T cells were defined as cells expressing 2 or 3 activation markers for the AIM assay and cells expressing IFNγ and/or IL2 for the ICS assay (see further AIM/ICS methods below). All samples with viability below 70% measured by flow cytometry or with a CD4 + or CD8 + T cell count below 10,000 were excluded from analyses.

### Intracellular cytokine stain assay

The ICS assay was performed essentially as previously published [38, 39]. For each sample, 4 conditions were used: DMSO, S peptide small pool, non-S peptide pool and staphylococcal enterotoxin B as a positive control. PBMCs were stimulated for 16–18 h at 37 °C, 5%CO2 in the presence of secretion inhibitors (Golgistop a final dilution of 1:2,000, and Golgiplug at a final dilution of 1:3,000, BD). After the stimulation, cells were stained with fixable Aqua dead cell stain (Invitrogen# L34966) as well as surface antibodies including CD14-PE-Cy5, CD19-PE-Cy5, CD56-PE-Cy5, CD3-BUV395, CD4-APC-fire750, CD8-BV786, CCR7-PE-dazzle594, CD45RA-Alexa700 and intracellular cytokine staining IFNγ and IL-2 using BD Cytofix/ Cytoperm protocol. Samples were acquired on a BD Fortessa X20 cytometer and data was analysed using FlowJo Version 10.8 using the following gating strategy: Live cells ➔ Single cells (FSC-A/FSC-H) ➔ Single cells (SSC-A/SSC-H) ➔ Lymphocytes (FSC-H/SSC-A) ➔ CD3 + T cells (CD14-CD19-CD56-/CD3 +) ➔ CD4 + or CD8 + T cells and then CCR7 versus CD45RA to define memory subsets within both the CD8 + and CD4 + T cell populations; naïve (CD45RA + CCR7 +), central memory (CM) (CD45RA-CCR7 +), effector memory (EM) (CD45RA-CCR7-) and terminally differentiated (TD) (CD45RA + CCR7-). For cytokine analyses, we defined a gate including all CD4 + /CD8 + memory and effector subsets (CM, EM and TD). Finally, within the CD4 + and CD8 + memory/effector subset, we gated IFNγ and IL2 and the frequency of cells expressing either IFNγ + IL2 + , IFNγ + IL2- or IFNγ-IL2 + was calculated by the “Boolean gating” function in FlowJo (Supp. Figure 2). The frequency of cytokine-producing antigen-specific T cells was determined by subtraction of the background cytokine response in unstimulated control samples from the positive response in the samples stimulated with SARS-CoV-2.

### Activation-induced marker assay (AIM assay)

The AIM assay was performed essentially as previously published [16, 40]. For each sample, 4 conditions were used: DMSO, S peptide large pool, S peptide small pool and non-S peptide pool. A subset of samples on each plate were stimulated with staphylococcal enterotoxin B (SEB, 1 μg/mL, #S4881) as a positive control. Following 20 h incubation at 37℃, supernatants were harvested for cytokine quantification and cells were stained with the following dye/antibodies: Fixable Near-IR dead cell stain (Invitrogen, #L34976), CD3-PerCP-Cy5.5 (Biolegend #344808), CD4-BV650 (Biolegend #300536), CD8-BV605 (Biolegend #301040), CD69-APC (Biolegend #310910), OX40-BV421 (Biolegend #350014), and 41BB-PE (Biolegend #309804). Samples were acquired on a MACSQuant16 and data was analysed using FlowJo 10.8 using the following gating strategy: Live cells ➔ Single cells (FSC-A/FSC-H) ➔ Lymphocytes (FSC-H/SSC-A) ➔ CD3 + T cells ➔ CD4 + or CD8 + T cells and the expression of CD69, OX40 and 41BB was determined within CD4 + /CD8 + T cells. The frequency of cells expressing either CD69 + OX40 + 4-1BB + , CD69 + OX40 + , CD69 + 4-1BB + or OX40 + 4-1BB + was calculated by the “Boolean gating” function in FlowJo (Supp. Figure 5). Lastly, the frequency of antigen-specific cells was determined by subtracting the frequency of the non-stimulation condition from each antigen-stimulated condition. Total SARS-CoV-2-specific cells were calculated as a summation of each of the 4 populations (CD69 + OX40 + 4-1BB + , CD69 + OX40 + 4-1BB-, CD69 + OX40-4-1BB + or CD69-OX40 + 4-1BB + T cells). For Figs. 2 and 4 we have defined a positive AIM response in an individual if the response was above 0.107% and 0.078% for SARS-CoV-2 specific CD4 + T cells and CD8 + T cells, respectively, as previously published. The threshold values of 0.107% and 0.078% (CD4 + T cells and CD8 + T cells, respectively) were calculated from the SARS-CoV-2 specific AIM response in SARS-CoV-2 negative individuals, that had not been vaccinated (*n* = 238) as follows: Threshold for a positive response = the median value plus one standard deviation (SD) [16].

### Cytokine detection in AIM assay

Interferon-γ (IFNγ) was measured in supernatants from the AIM assay using Meso Scale Discovery (MSD) platform (Meso Scale Diagnostics LLC, Maryland, USA) V-PLEX Plus Proinflammatory Panel 1 (K15049G) according to the manufacturer’s instructions at a dilution of 1:200. Plates were read on a MESO QuickPlex SQ 120 reader. Raw data were processed by Discovery Workbench 4.0 Software. Quantifications were reported in pg/mL. To investigate the antigen-specific IFNγ response, the measurement from the non-stimulated condition was subtracted from the antigen-stimulated condition.

### Data and statistical analysis

Data were shown as individual points with a boxplot indicating median and IQR, and error bars showing 95% CI for the longitudinal study and connected individual points for the vaccine-induced immunity. To assess the statistical change over time both Kruskal–Wallis and Friedman’s test was used to check for differences between visits. Kruskal–Wallis was used on the whole dataset and to account for measurements being repeated, Friedman test was used excluding participant data where the analysis was not quantifiable for one or more visits. Post hoc analysis was done using Wilcoxon rank-sum adjusted using Bonferroni with visit 1 as a reference. Wilcoxon signed-rank test was used to compare the vaccine-induced immune response. *P* values ≤ 0.05 were considered statistically significant. *P* values were denoted as follows: * = *p* ≤ 0.05, ** = *p* < 0.01, *** = *p* < 0.001, and **** = *p* < 0.0001.

Data analysis and visualization was conducted in R (version 4.2.2), and RStudio Desktop (version 2022.12.0 + 353).

### Supplementary Information

**Additional file 1: Supplementary Figure 1.** T cell memory subsets within overall CD4+ and CD8+ T cells. (A) Composition of memory subsets within the overall CD4+ and CD8+ T cells during visits 1,3,4 and 5. Memory subsets were defined as naïve (CD45RA+CCR7+), central memory (CM) (CD45RA-CCR7+), effector memory (EM) (CD45RA-CCR7-) and terminally differentiated (TD) (CD45RA+CCR7-). Individual values and box and whisker plots with median values shown (Box shows IQR, error bars indicate 95% CI). (B) Stacked bar graphs illustrate the movement of memory subsets within the CD4+ and CD8+ T cells during visits 1,3,4 and 5. (A-B).

**Additional file 2: Supplementary Figure 2.** Gating strategy for flow cytometric intracellular cytokine staining(ICS). (A-C) Shown are dot plots for patient 9 at visit 1. (B) Response to stimulation with negative control (DMSO) and (C) SARS-CoV-2 -peptide pools for effector/memory CD4^+^ T cells and effector/memory CD8^+^ T cells. Numbers represent percentage of the shown population that’s within the shown gate. (D) Frequency of cells expressing only one or two cytokines (IFNγ+IL2+, IFNγ+IL2- or IFNγ-IL2+) calculated by the Boolean gating.

**Additional file 3: Supplementary Figure 3.** IL4 production by SARS-CoV-2 specific cells. IL4 production by SARS-CoV-2 specific cells measured in the supernatant harvested from the cell stimulations in the AIM assay (Analysed by mesoscale) after stimulation with (A) Non-spike peptide pool, (B), Spike-small pool or (C) Spike-large pool. Horizontal line shows median Statistical comparisons were performed using Wilcoxon unpaired signed-ranks test adjusted using Bonferroni. *P ≤ 0.05, **P < 0.01, ***P < 0.001, ****P < 0.0001, no asterisk indicates non-significance.

**Additional file 4: Supplementary Figure 4.** Impact of SARS-CoV-2 vaccination on humoral and cellular immunity for S-small and non-S immunity. Analysis of a subset of 65 patients who received their first 2 vaccinations between two subsequent visits (Prior: A visit where the participant had not been vaccinated, Post: The subsequent visit, where the participant had received 2 vaccinations). (A) Percentage of SARS-CoV-2 spike (S)-specific CD4+ and CD8+ T cells analysed by AIM after stimulation with the S-small pool. (B) IFNγ production by SARS-CoV-2 S-specific cells (S-small pool). (C) SARS-CoV-2 nucleocapsid-specific IgG levels (D) Percentage of SARS-CoV-2 S-specific CD4+ and CD8+ memory T cells analysed by ICS after stimulation with the non-S peptide pool. (E) Percentage of SARS-CoV-2 non-S-specific CD4+ and CD8+ T cells analysed by AIM. (F) IFNγ production by SARS-CoV-2 non-S-specific cells. Horizontal line shows median Statistical comparisons were performed using Wilcoxon unpaired signed-ranks test adjusted using Bonferroni. *P ≤ 0.05, **P < 0.01, ***P < 0.001, ****P < 0.0001.

**Additional file 5: Supplementary Figure 5.** Gating strategy for flow activation induced marker assay (AIM). (A-C) Shown are dot plots for patient 9 at visit 1. (B) Response to stimulation with negative control (DMSO) and (C) SARS-CoV-2 -peptide pools for CD4^+^ T cells and CD8^+^ T cells. Numbers represent percentage of the shown population that's within the shown gate. (D) Frequency of cells expressing two or three activation markers (CD69+OX40+4-1BB+, CD69+OX40+, CD69+4-1BB+ or OX40+4-1BB+) calculated by the Boolean gating.

**Additional file 6: Supplementary Table 1.** Vaccine information and time from PCR to visit.

**Additional file 7: Supplementary Table 2.** Vaccine response.

**Additional file 8: Supplementary Table 3.** Peptides in the Spike peptide small pool and the Non-spike peptide pool.
