# Supplementary figures and images for: Long-term humoral and cellular immunity after primary SARS-CoV-2 infection: a 20-month longitudinal study
Source: BMC Immunol. 2023 Nov 16;24:45. doi: 10.1186/s12865-023-00583-y (PMC10652616; doi:10.1186/s12865-023-00583-y)

(a)

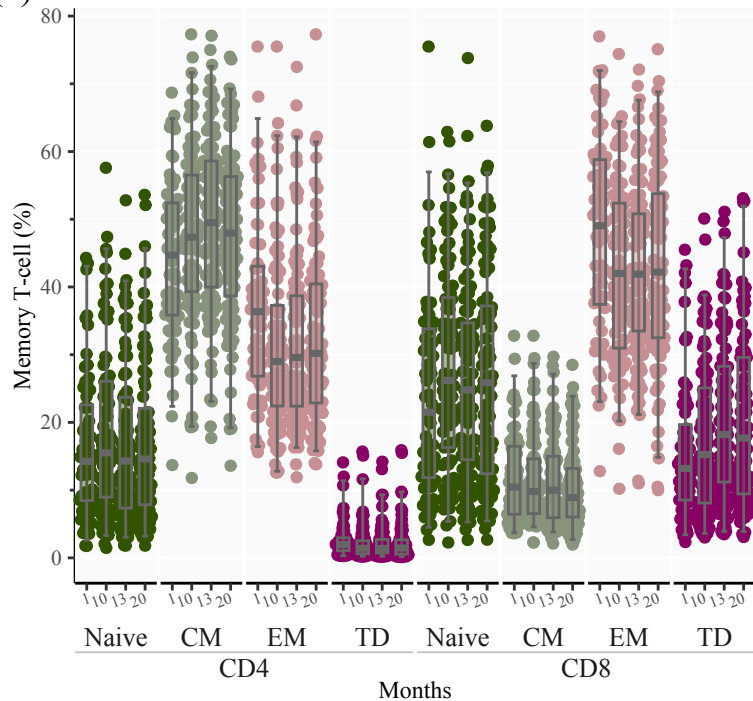

(b)

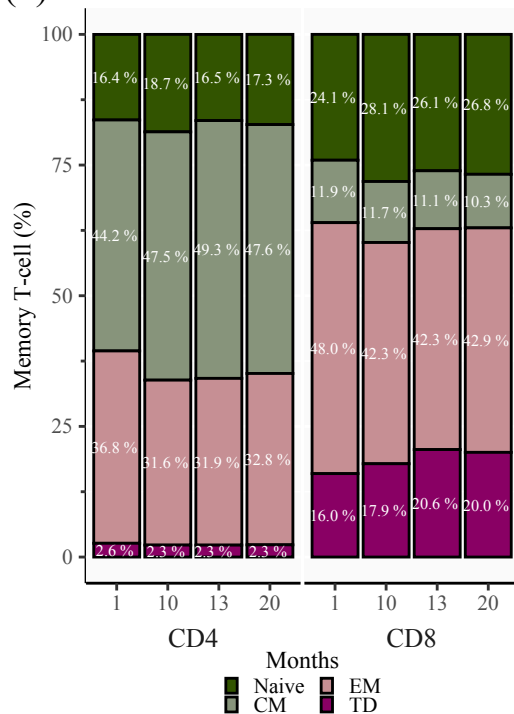

Supplement: Supplementary file 1 — Additional file 1: Supplementary Figure 1. T cell memory subsets within overall CD4+ and CD8+ T cells. (A) Composition of memory subsets within the overall CD4+ and CD8+ T cells during visits 1,3,4 and 5. Memory subsets were defined as naïve (CD45RA+CCR7+), central memory (CM) (CD45RA-CCR7+), effector memory (EM) (CD45RA-CCR7-) and terminally differentiated (TD) (CD45RA+CCR7-). Individual values and box and whisker plots with median values shown (Box shows IQR, error bars indicate 95% CI). (B) Stacked bar graphs illustrate the movement of memory subsets within the CD4+ and CD8+ T cells during visits 1,3,4 and 5. (A-B). [file 12865_2023_583_MOESM1_ESM.pdf]

# IL4 measured in supernatant from AIM

(a)

Non-spike

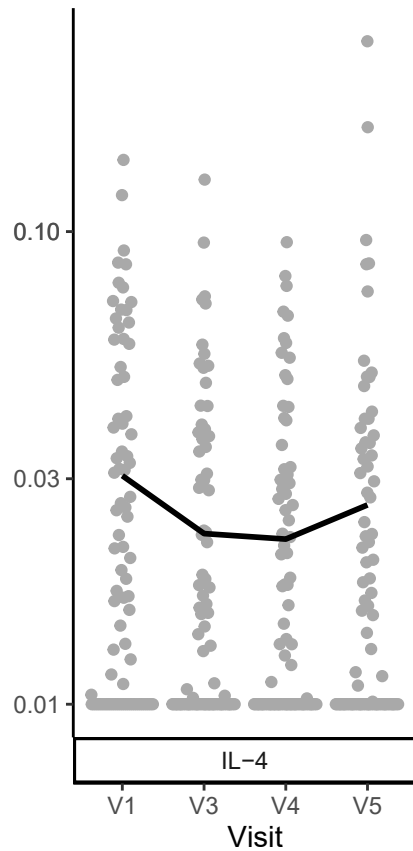

(b)

Small-spike

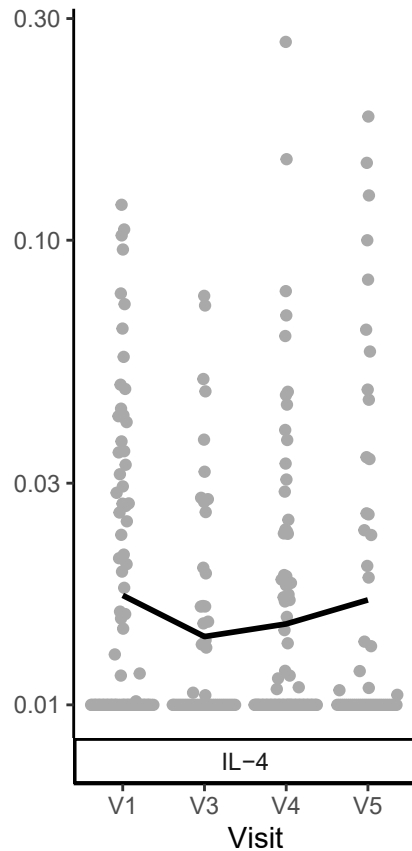

(c)

Large-spike

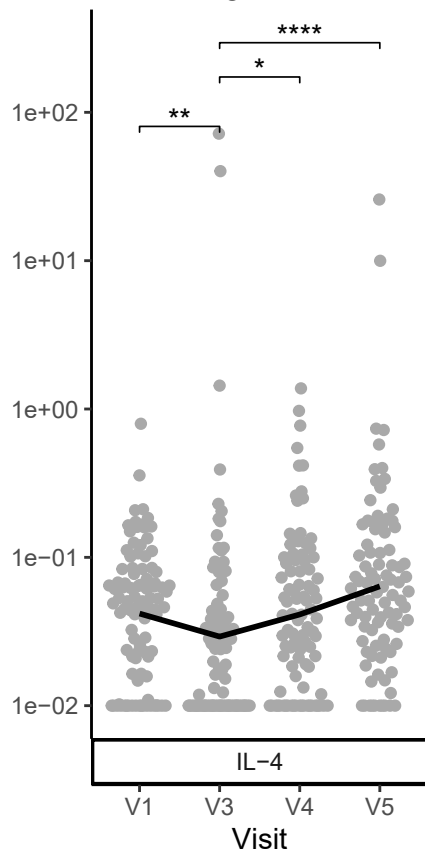

Supplement: Supplementary file 3 — Additional file 3: Supplementary Figure 3. IL4 production by SARS-CoV-2 specific cells. IL4 production by SARS-CoV-2 specific cells measured in the supernatant harvested from the cell stimulations in the AIM assay (Analysed by mesoscale) after stimulation with (A) Non-spike peptide pool, (B), Spike-small pool or (C) Spike-large pool. Horizontal line shows median Statistical comparisons were performed using Wilcoxon unpaired signed-ranks test adjusted using Bonferroni. *P ≤ 0.05, **P < 0.01, ***P < 0.001, ****P < 0.0001, no asterisk indicates non-significance. [file 12865_2023_583_MOESM3_ESM.pdf]

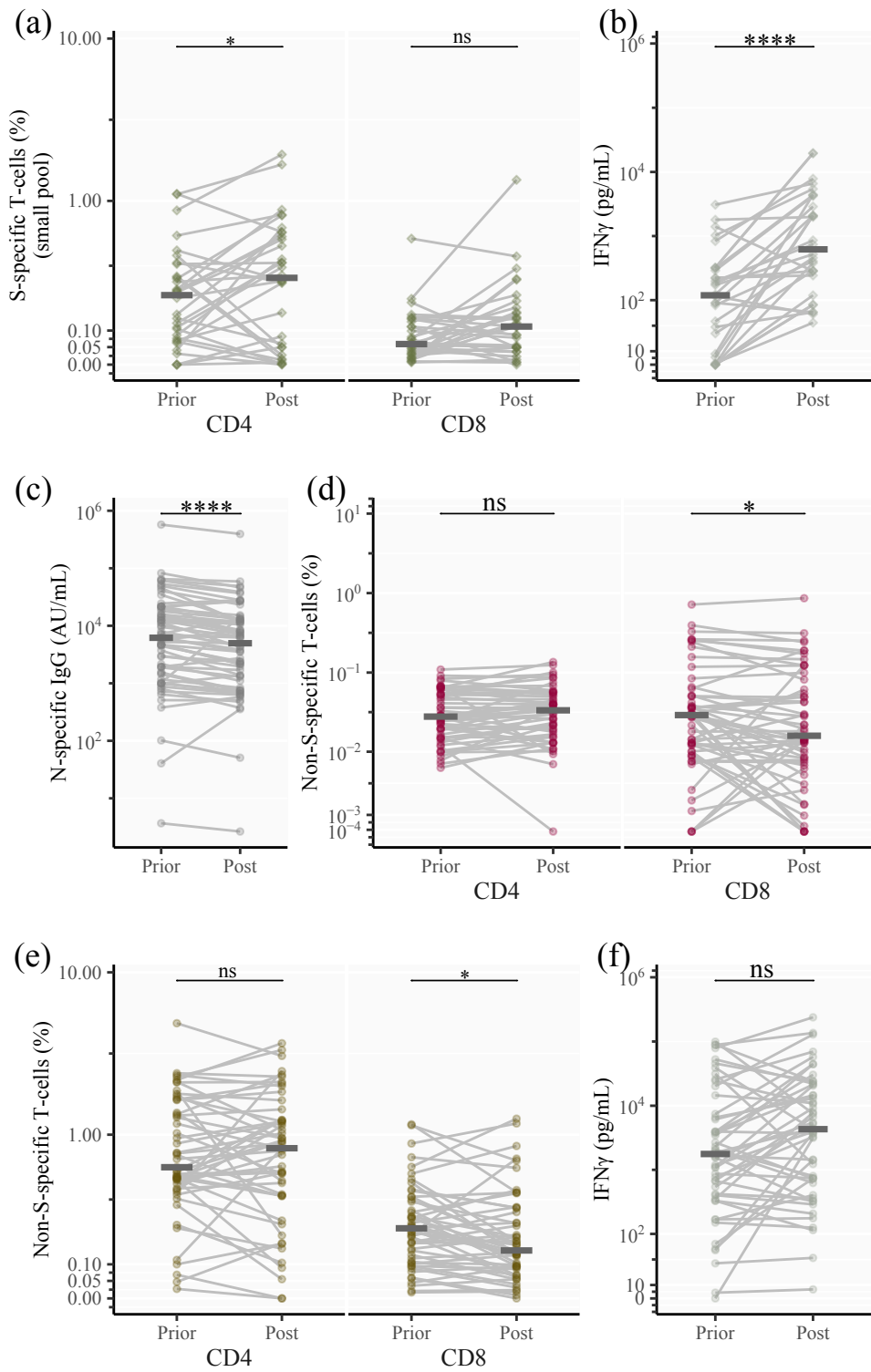

Supplement: Supplementary file 4 — Additional file 4: Supplementary Figure 4. Impact of SARS-CoV-2 vaccination on humoral and cellular immunity for S-small and non-S immunity. Analysis of a subset of 65 patients who received their first 2 vaccinations between two subsequent visits (Prior: A visit where the participant had not been vaccinated, Post: The subsequent visit, where the participant had received 2 vaccinations). (A) Percentage of SARS-CoV-2 spike (S)-specific CD4+ and CD8+ T cells analysed by AIM after stimulation with the S-small pool. (B) IFNγ production by SARS-CoV-2 S-specific cells (S-small pool). (C) SARS-CoV-2 nucleocapsid-specific IgG levels (D) Percentage of SARS-CoV-2 S-specific CD4+ and CD8+ memory T cells analysed by ICS after stimulation with the non-S peptide pool. (E) Percentage of SARS-CoV-2 non-S-specific CD4+ and CD8+ T cells analysed by AIM. (F) IFNγ production by SARS-CoV-2 non-S-specific cells. Horizontal line shows median Statistical comparisons were performed using Wilcoxon unpaired signed-ranks test adjusted using Bonferroni. *P ≤ 0.05, **P < 0.01, ***P < 0.001, ****P < 0.0001. [file 12865_2023_583_MOESM4_ESM.pdf]
