# Supplementary material for: Long-term humoral and cellular immunity after primary SARS-CoV-2 infection: a 20-month longitudinal study
Source: BMC Immunol. 2023 Nov 16;24:45. doi: 10.1186/s12865-023-00583-y (PMC10652616; doi:10.1186/s12865-023-00583-y)
Supplement: Supplementary file 2 — Additional file 2: Supplementary Figure 2. Gating strategy for flow cytometric intracellular cytokine staining(ICS). (A-C) Shown are dot plots for patient 9 at visit 1. (B) Response to stimulation with negative control (DMSO) and (C) SARS-CoV-2 -peptide pools for effector/memory CD4+ T cells and effector/memory CD8+ T cells. Numbers represent percentage of the shown population that’s within the shown gate. (D) Frequency of cells expressing only one or two cytokines (IFNγ+IL2+, IFNγ+IL2- or IFNγ-IL2+) calculated by the Boolean gating. [file 12865_2023_583_MOESM2_ESM.pdf]

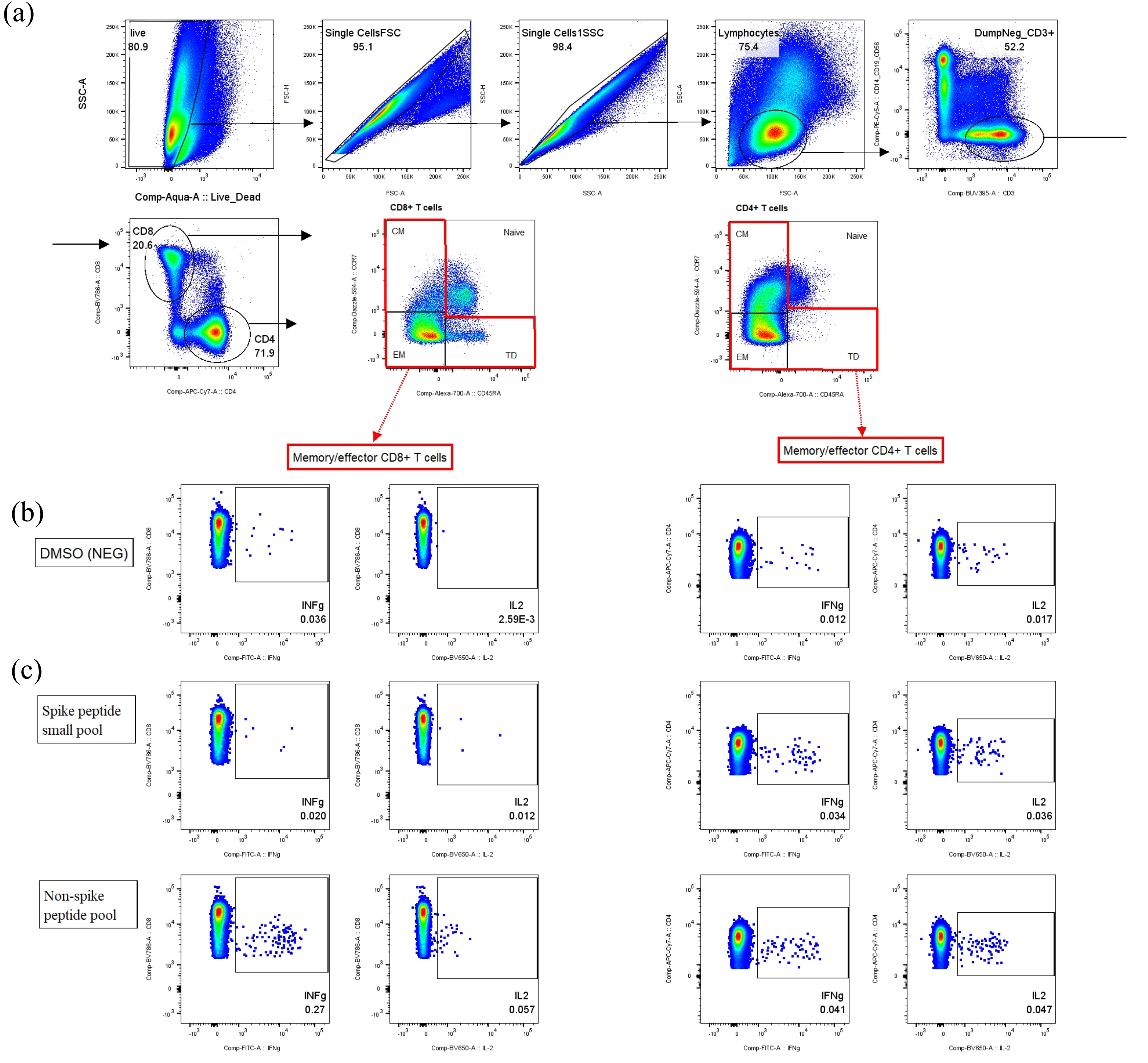

(d)

Memory/effector CD8+ T cells: Boolean combination gates			
	IFNγ+IL2+	IFNγ-IL2+	IFNγ+IL2-
DMSO	0·003	0·00	0·034
Spike - small	0·006	0·006	0·015
Non - spike	0·052	0·005	0·210

Memory/effector CD4+ T cells: Boolean combination gates			
	IFNγ+IL2+	IFNγ-IL2+	IFNγ+IL2-
DMSO	0·006	0·011	0·006
Spike - small	0·02	0·016	0·014
Non - spike	0·024	0·024	0·018
