# Supplementary material for: Long-term humoral and cellular immunity after primary SARS-CoV-2 infection: a 20-month longitudinal study
Source: BMC Immunol. 2023 Nov 16;24:45. doi: 10.1186/s12865-023-00583-y (PMC10652616; doi:10.1186/s12865-023-00583-y)
Supplement: Supplementary file 5 — Additional file 5: Supplementary Figure 5. Gating strategy for flow activation induced marker assay (AIM). (A-C) Shown are dot plots for patient 9 at visit 1. (B) Response to stimulation with negative control (DMSO) and (C) SARS-CoV-2 -peptide pools for CD4+ T cells and CD8+ T cells. Numbers represent percentage of the shown population that's within the shown gate. (D) Frequency of cells expressing two or three activation markers (CD69+OX40+4-1BB+, CD69+OX40+, CD69+4-1BB+ or OX40+4-1BB+) calculated by the Boolean gating. [file 12865_2023_583_MOESM5_ESM.pdf]

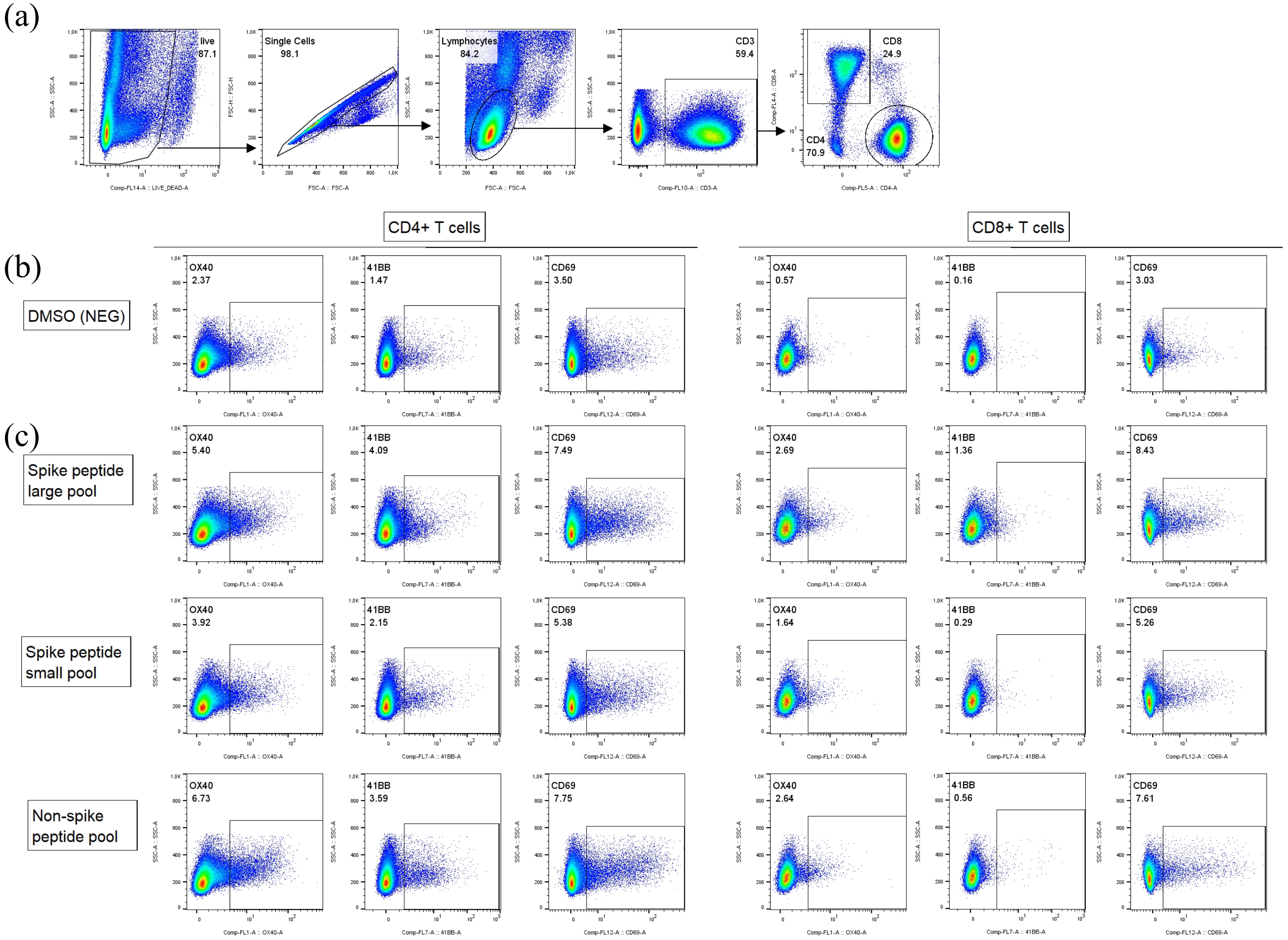

(d)

CD4+ T cells: Boolean combination gates				
	41BB+CD69+ OX40+	41BB+CD69+ OX40-	41BB+CD69- OX40+	41BB-CD69+ OX40+
DMSO	0·25	0·065	0·35	0·76
Spike - large	0·53	0·2	0·75	2·25
Spike - small	0·39	0·076	0·65	1·51
Non - spike	0·86	0·13	1·13	2·91

CD8+ T cells: Boolean combination gates				
	41BB+CD69+ OX40+	41BB+CD69+ OX40-	41BB+CD69- OX40+	41BB-CD69+ OX40+
DMSO	0·019	0·052	0·0032	0·071
Spike - large	0·091	0·39	0·038	0·93
Spike - small	0·041	0·14	0·01	0·45
Non - spike	0·11	0·31	0·0036	0·8
