# Supplementary material for: Long-term humoral and cellular immunity after primary SARS-CoV-2 infection: a 20-month longitudinal study
Source: BMC Immunol. 2023 Nov 16;24:45. doi: 10.1186/s12865-023-00583-y (PMC10652616; doi:10.1186/s12865-023-00583-y)
Supplement: Supplementary file 6 — Additional file 6: Supplementary Table 1. Vaccine information and time from PCR to visit. [file 12865_2023_583_MOESM6_ESM.docx]

**Supplementary table 1 – Vaccine information and time from PCR to visit**

|  | N = 93 |
| --- | --- |
| Days from PCR to visit, Median [IQR] | |
| V1 | 41 [34, 45] |
| V3 | 310 [304, 317] |
| V4 | 411 [403, 415] |
| V5 | 608 [598, 614] |
|  | |
| Vaccine, n (%) |  |
| AstraZeneca | 1 (1.1%) |
| AstraZeneca + Pfizer-BioNTech | 6 (6.5%) |
| Johnson & Johnson | 3 (3.3%) |
| Moderna | 11 (12%) |
| Pfizer-BioNTech | 71 (77%) |
| Number of vaccines received | |
| Visit 1, n (%) |  |
| 0 Doses | 93 (100) |
| Visit 3, n (%) |  |
| 0 Doses | 84 (90) |
| 1 Dose | 9 (9.7) |
| Visit 4, n (%) |  |
| 0 Doses | 62 (67) |
| 1 Dose | 12 (13) |
| 2 Doses | 19 (20) |
| Visit 5, n (%) |  |
| 0 Doses | 1 (1.1) |
| 1 Dose | 6 (6.5) |
| 2 Doses | 69 (74) |
| 3 Doses | 17 (18) |
