# Supplementary material for: Long-term humoral and cellular immunity after primary SARS-CoV-2 infection: a 20-month longitudinal study
Source: BMC Immunol. 2023 Nov 16;24:45. doi: 10.1186/s12865-023-00583-y (PMC10652616; doi:10.1186/s12865-023-00583-y)
Supplement: Supplementary file 7 — Additional file 7: Supplementary Table 2. Vaccine response. [file 12865_2023_583_MOESM7_ESM.docx]

**Supplementary table 2 – Vaccine response**

| No of Doses | 2 Doses, N = 65 |
| --- | --- |
| Days from vaccine to visit, Median [IQR] | 112 [92, 130] |
| Sex, n (%) |  |
| Female | 24 (37%) |
| Male | 41 (63%) |
| Visit, n (%) |  |
| V4 | 10 (15%) |
| V5 | 55 (85%) |
| Vaccine, n (%) |  |
| Johnson & Johnson | 1 (1.5%) |
| Moderna | 10 (15%) |
| Pfizer-BioNTech | 54 (83%) |
