# Supplementary material for: Long-term humoral and cellular immunity after primary SARS-CoV-2 infection: a 20-month longitudinal study
Source: BMC Immunol. 2023 Nov 16;24:45. doi: 10.1186/s12865-023-00583-y (PMC10652616; doi:10.1186/s12865-023-00583-y)
Supplement: Supplementary file 8 — Additional file 8: Supplementary Table 3. Peptides in the Spike peptide small pool and the Non-spike peptide pool. [file 12865_2023_583_MOESM8_ESM.docx]

**Supplementary table 3 - Peptides in the Spike peptide small pool and the Non-spike peptide pool**

| **Pool** | **Peptide** | **Sequence** | **Parent protein** | **Protein class** |
| --- | --- | --- | --- | --- |
| **Spike peptide small pool** | 1 | ITRFQTLLALHRSYL | ORF2 spike | structural |
|  | 2 | QYIKWPWYI | ORF2 spike | structural |
|  | 3 | LTDEMIAQY | ORF2 spike | structural |
|  | 4 | SPRRARSVA | ORF2 spike | structural |
|  | 5 | YEQYIKWPW | ORF2 spike | structural |
|  | 6 | YLQPRTFLL | ORF2 spike | structural |
|  | 7 | KCYGVSPTK | ORF2 spike | structural |
|  | 8 | CTFEYVSQPFLMDLE | ORF2 spike | structural |
|  | 9 | NLLLQYGSFCTQLNR | ORF2 spike | structural |
|  | 10 | TDEMIAQYTSALLAG | ORF2 spike | structural |
|  | 11 | LLALHRSYL | ORF2 spike | structural |
| **Non-spike peptide pool** | 12 | LDDFVEIIKSQDLSV | ORF1 | non-structural |
|  | 13 | TTDPSFLGRY | ORF1 | non-structural |
|  | 14 | VYIGDPAQL | ORF1 | non-structural |
|  | 15 | IEYPIIGDEL | ORF1 | non-structural |
|  | 16 | DLKGKYVQI | ORF1 | non-structural |
|  | 17 | ILFTRFFYV | ORF1ab | non-structural |
|  | 18 | STFNVPMEK | ORF1ab | non-structural |
|  | 19 | ASMPTTIAK | ORF1ab | non-structural |
|  | 20 | INVFAFPFTIYSLL | ORF10 | accessory |
|  | 21 | FMRIFTIGTVTLKQG | ORF3 | accessory |
|  | 22 | VYFLQSINF | ORF3 | accessory |
|  | 23 | ALSKGVHFV | ORF3 | accessory |
|  | 24 | FTSDYYQLY | ORF3A | accessory |
|  | 25 | LLYDANYFL | ORF3A | accessory |
|  | 26 | FYVYSRVKNLNSSRV | ORF4 env | structural |
|  | 27 | LSYYKLGASQRVAGD | ORF5 mem | structural |
|  | 28 | SELVIGAVIL | ORF5 MEM | structural |
|  | 29 | IWNLDYIINLIIKNL | ORF6 | accessory |
|  | 30 | QEEVQELYSPIFLIV | ORF7 | accessory |
|  | 31 | QLRARSVSPK | ORF7 | accessory |
|  | 32 | LEYHDVRVVL | ORF8 | accessory |
|  | 33 | KDGIIWVATEGALNT | ORF9 nuc | structural |
|  | 34 | GTWLTYTGAIKLDDK | ORF9 nuc | structural |
|  | 35 | RWYFYYLGTGPEAGL | ORF9 nuc | structural |
|  | 36 | LLLLDRLNQLESKMS | ORF9 nuc | structural |
|  | 37 | ASWFTALTQHGKEDL | ORF9 nuc | structural |
|  | 38 | IGYYRRATRRIRGGD | ORF9 nuc | structural |
|  | 39 | ASAFFGMSRIGMEVT | ORF9 nuc | structural |
|  | 40 | ATEGALNTPK | ORF9 nuc | structural |
|  | 41 | MEVTPSGTWL | ORF9 nuc | structural |
|  | 42 | KTFPPTEPKK | ORF9 nuc | structural |
|  | 43 | LLLLDRLNQL | ORF9 nuc | structural |
|  | 44 | QRNAPRITF | ORF9 nuc | structural |
|  | 45 | SPRWYFYYL | ORF9 nuc | Structural |

**Supplementary table 3 - Peptides in the Spike peptide small pool and the Non-spike peptide pool**

Peptides included in the Spike peptide small pool (S-small) consisting of 11 immunodominant S peptides (JPT peptides, custom order)(utilized in AIM/ICS) and peptides included in the non-Spike peptide pool (non-S) consisting of 34 immunodominant peptides outside the S protein (JPT peptides, custom order) )(utilized in AIM/ICS).
